# Formation-Dependent Cardinality Concealment of Target Swarms in Multifunction Radar

**DOI:** 10.3390/s26206644

**Published:** 2026-10-21

**Authors:** Geon Lee, Seong-Ryul Park, Hyun-Dong Kim, Seung-Hwan Kim, Nam-Hoon Jeong, Kyung-Tae Kim

**Affiliations:** 1 Department of Electrical Engineering, Pohang University of Science and Technology, 77 Cheongam-ro, Nam-gu, Pohang 37673, Republic of Korea; glee72@postech.ac.kr (G.L.);; 2 Agency for Defense Development, Yuseong P.O. Box 35, Daejeon 34186, Republic of Korea

**Keywords:** multifunction radar, cardinality estimation, radar resource management, unmanned surface vehicle

## Abstract

Recognizing a swarm of unmanned surface vehicles does not guarantee that its physical cardinality can be recovered. This study examines how formation, viewing aspect ψ, defined as the orientation of the formation relative to the radar line of sight (RLOS), and sensing resources affect these two tasks in a multifunction radar. A two-stage simulation framework evaluates group recognition against a single extended target (Stage 1) and cardinality recovery (Stage 2) using regularized linear discriminant analysis, with the aggregate nominal radar cross-section held constant. Certification is assessed using one-sided 95% confidence bounds. At *N* = 9 and baseline resources, group observability is certified in 12 of 35 formation–aspect conditions. For abreast formations at $\psi =60^{\circ }$ and $90^{\circ }$, the group selection probability is 0.993, whereas the exact recovery probabilities are 0.108 and 0.067. This cardinality concealment, in which group observability is certified but exact recovery is not, persists over N=6,…,12 for both geometries. Coarse size information nevertheless remains, as 52–56% of estimates over N=3,…,12 lie within one target of the true value, against 28% for uniform guessing. Increasing bandwidth up to tenfold or coherent dwell up to fourfold yields no certified exact recovery. These results are specific to the evaluated simulation model and signal-processing framework and do not establish a general limitation of radar systems.

## 1. Introduction

Radar has long served as a primary surveillance sensor owing to its ability to provide long-range coverage with relative insensitivity to illumination conditions and adverse weather [1]. The development of phased-array and active electronically scanned array (AESA) technology has enabled rapid electronic beam steering and flexible control of radar tasks without relying solely on mechanical scanning [1]. Building on this flexibility, modern multifunction radars (MFRs) integrate multiple functions, including surveillance, detection, tracking, and target classification, within a single radar system [2,3]. These functions, however, compete for finite radar resources associated with time, energy, frequency, and processing capacity. Consequently, allocating additional dwell time, waveform resources, or repeated observations to one target reduces the resources available to other concurrent tasks, making radar resource management an essential component of MFR operation [2,3,4].

In recent years, advances in autonomous navigation, cooperative control, and low-cost unmanned platforms have made it increasingly feasible to operate multiple unmanned surface vehicles (USVs) in coordinated or swarm configurations [5,6,7,8]. The simultaneous presence of many relatively low-cost platforms can increase the surveillance, tracking, and response burden on a defending system. Under such conditions, the information required from a radar extends beyond the conventional question of whether a target is present. The radar must first determine whether an observed return originates from a single target or from a group of targets and, once a swarm is identified, estimate how many individual platforms constitute it. This distinction is operationally important because the estimated swarm size can affect threat assessment, track management, and the allocation of subsequent sensing or response resources. In maritime environments, this inference is further complicated by wave-induced vessel motion and sea clutter, which can alter the Doppler and amplitude structure of small surface-target returns [9,10,11].

A USV swarm may produce radar signatures that differ substantially from those of a single target because of both the individual motions of its constituent platforms and their relative spatial arrangement. In particular, these signatures depend strongly on the formation adopted by the swarm and on the aspect from which it is observed. Even for an identical number of platforms, different formation–aspect combinations can project different structures into the radar range–angle–Doppler space. In favorable geometries, multiple members may occupy separable range, angular, or Doppler resolution cells, whereas in unfavorable geometries, their responses can overlap and form a composite return similar to that of a single extended target. Related ambiguity between partly unresolved target groups and extended objects has previously been recognized in extended and group target tracking [12]. The present study examines this ambiguity from a sensing perspective by considering how formation and viewing aspect affect the information available for distinguishing a swarm from a single extended target and for estimating its physical cardinality. The ability to recognize that a return originates from a swarm does not necessarily imply that its physical cardinality can also be inferred. In this work, we use the term cardinality concealment to denote an operational condition under the prescribed evaluation protocol in which group observability is certified while exact cardinality recovery is not certified. The term concealment refers to radar observability under this protocol and does not imply deliberate deceptive behavior by the swarm.

Existing approaches address different aspects of the target cardinality estimation problem. Source enumeration methods estimate the number of signal components using criteria such as the Akaike information criterion (AIC) and minimum description length (MDL), with continued efforts addressing closely spaced or coherent sources [13,14,15,16,17]. Radar studies of unresolved targets examine whether multiple returns can be distinguished under limited resolution [18,19], while random finite set methods estimate target cardinality in the presence of uncertain detections and data association [20]. Extended and group target tracking methods instead represent spatially distributed or collectively moving targets after measurements have been formed [12,21,22,23]. The present work examines whether the radar measurement contains sufficient structure to distinguish a swarm from a single extended target and, once a group return is present, to recover the physical number of constituent platforms. It further examines how these two levels of observability vary with projected geometry and sensing resources.

Among the controllable sensing dimensions of an MFR, waveform bandwidth and coherent dwell provide two physically interpretable mechanisms for increasing measurement resolution. Increasing the transmitted bandwidth *B* improves range resolution according to ΔR=c/(2B) [24], potentially separating platforms whose projections along the line of sight overlap at the baseline bandwidth. Increasing the number of coherently processed pulses $N_{p}$ extends the coherent processing interval (CPI) and improves Doppler resolution, potentially revealing differences in radial velocity among swarm members [24]. These improvements consume radar resources associated with dwell time, waveform allocation, revisit scheduling, and transmitted energy that must be shared among competing surveillance and tracking functions [2,3].

Motivated by this problem, this paper develops a two-stage framework for evaluating radar observability of target swarms. Stage 1 first discriminates a target swarm from a single extended target at the reference cardinality $N_{\mathrm{ref}}=9$ and evaluates group observability as a function of formation and viewing aspect. It is then extended over N∈{3,…,12} for four representative geometries to identify the cardinality ranges over which group observability is certified. Stage 2 estimates the physical number of swarm members over the same cardinality range and evaluates the probability of exact cardinality recovery. The two stages are compared under the baseline sensing resources to identify conditions in which group observability and exact cardinality recovery diverge, and Stage 2 further examines how exact cardinality recovery changes as bandwidth and coherent dwell are increased.

This paper makes three contributions. First, it formulates separate confidence-bound certification criteria for group recognition and exact cardinality recovery under a controlled aggregate radar cross-section (RCS) condition. Second, it develops a two-stage framework for examining the dependence of group and cardinality observability on formation, viewing aspect, and physical cardinality. Third, it evaluates the effects of bandwidth and coherent dwell on cardinality recovery and examines intra-target fragmentation as a potential mechanism underlying the observed resource dependence.

## 2. Signal and Scenario Model

### 2.1. Radar and Sensing Resource Model

A pulse–Doppler MFR observing a maritime target swarm is considered. The array configuration, operating wavelength λ, pulse repetition frequency (PRF), and the corresponding pulse repetition interval $T_{\mathrm{PRI}}$ are held fixed throughout. The sensing-resource analysis varies the waveform bandwidth *B* and the number of coherently processed pulses $N_{p}$ independently. The native range and Doppler resolutions and the coherent processing interval are updated accordingly, while the array configuration, wavelength, PRF, scenario parameters, and per-pulse signal-to-noise ratio (SNR) and clutter-to-noise ratio (CNR) are held fixed.

The nominal range resolution and the coherent processing quantities are

(1) $$\Delta R=\frac{c}{2B},$$

and

(2) $$\begin{array}{cc} T_{\mathrm{CPI}} & =N_{p}T_{\mathrm{PRI}},\\ \Delta f_{D} & \approx \frac{1}{T_{\mathrm{CPI}}},\\ \Delta v & =\frac{\lambda }{2T_{\mathrm{CPI}}}. \end{array}$$

The baseline sensing condition is denoted by $\left(B_{0},N_{p,0}\right)$. Increasing *B* improves native range resolution, while increasing $N_{p}$ extends the coherent processing interval and improves Doppler resolution. In the implemented $N_{p}$ sweep, the target and noise levels of each pulse are held fixed. Increasing $N_{p}$ therefore also increases the energy available over the coherent processing interval, so the $N_{p}$ sweep represents an increase in coherent observation resource that combines finer Doppler resolution with additional observation energy. The radar, scenario, processing, and evaluation parameters are summarized in Table 1. In the table, CFAR, OS-CFAR, and PCA denote constant false alarm rate, ordered-statistic CFAR, and principal component analysis, respectively.

### 2.2. Swarm Scenario and Formation Geometry

A maritime target swarm consisting of *N* surface targets is considered at centroid range $R_{c}$ and nominal centroid velocity $v_{c}$. The tested cardinality range is N∈{3,…,12}. The abreast, column, grid, and wedge formations use a fixed nominal inter-member spacing of s=50 m. The disk formation is generated by sampling uniformly over the area of a circular support of radius $R_{\mathrm{disk}}=150$ m subject to a minimum inter-target separation of $d_{\min}=15$ m. These fixed geometric scales provide controlled comparisons across cardinalities.

Let $p_{c}$ denote the position of the swarm centroid in the radar coordinate system, and let $q_{k}\left(F,N;\xi_{F}\right)$ denote the position of the *k*th target relative to the centroid in a coordinate system fixed to the formation, where *F* denotes the formation type, and $\xi_{F}$ denotes its geometric parameters specific to the formation. The formation set considered in the swarm observability analysis is

(3) F={abreast,column,grid,disk,wedge}.

To account for viewing geometry, the formation is rotated by the aspect angle ψ with respect to the radar line of sight (RLOS). The position of the *k*th target in the radar coordinate system is expressed as

(4) $$p_{k}=p_{c}+R\left(\psi \right)q_{k}\left(F,N;\xi_{F}\right),\;k=1,\ldots ,N,$$

where R(ψ) is the two-dimensional rotation matrix

(5) $$R\left(\psi \right)=\left[\begin{array}{cc} \cos\psi & -\sin\psi \\ \sin\psi & \cos\psi \end{array}\right].$$

Equation (4) shows that the radar-relative geometry depends jointly on the physical formation and viewing aspect. Consequently, the same physical cardinality can produce substantially different projections along the range and angular dimensions even when the formation itself is unchanged. For example, the nominal abreast formation with N=9 and s=50m has a total transverse extent of 400m. At $\psi =0^{\circ }$, this extent lies predominantly in the cross-range direction, and spherical range curvature at $R_{c}=10\;\mathrm{km}$ produces only about 2.0m of slant-range depth. In contrast, at $\psi =90^{\circ }$, the same physical extent is projected predominantly along the RLOS and spans approximately 26.7 baseline range-resolution cells. Thus, the viewing aspect redistributes the same physical formation across the dimensions observable by the radar.

Nominally different formation labels can also produce equivalent radar-relative geometries under rotation. For example, the nominal abreast formation at $\psi =90^{\circ }$ is geometrically equivalent, up to member ordering, to the nominal column formation at $\psi =0^{\circ }$. This rotational equivalence provides a physical consistency condition for the evaluation pipeline and is examined explicitly in Section 4.3.

Denoting the RLOS and cross-range coordinates of the *k*th target relative to the radar by $r_{k}$ and $x_{k}$, respectively, its angular position is expressed as

(6) $$\theta_{k}=\arctan\left(\frac{x_{k}}{r_{k}}\right).$$

**Figure 1:**
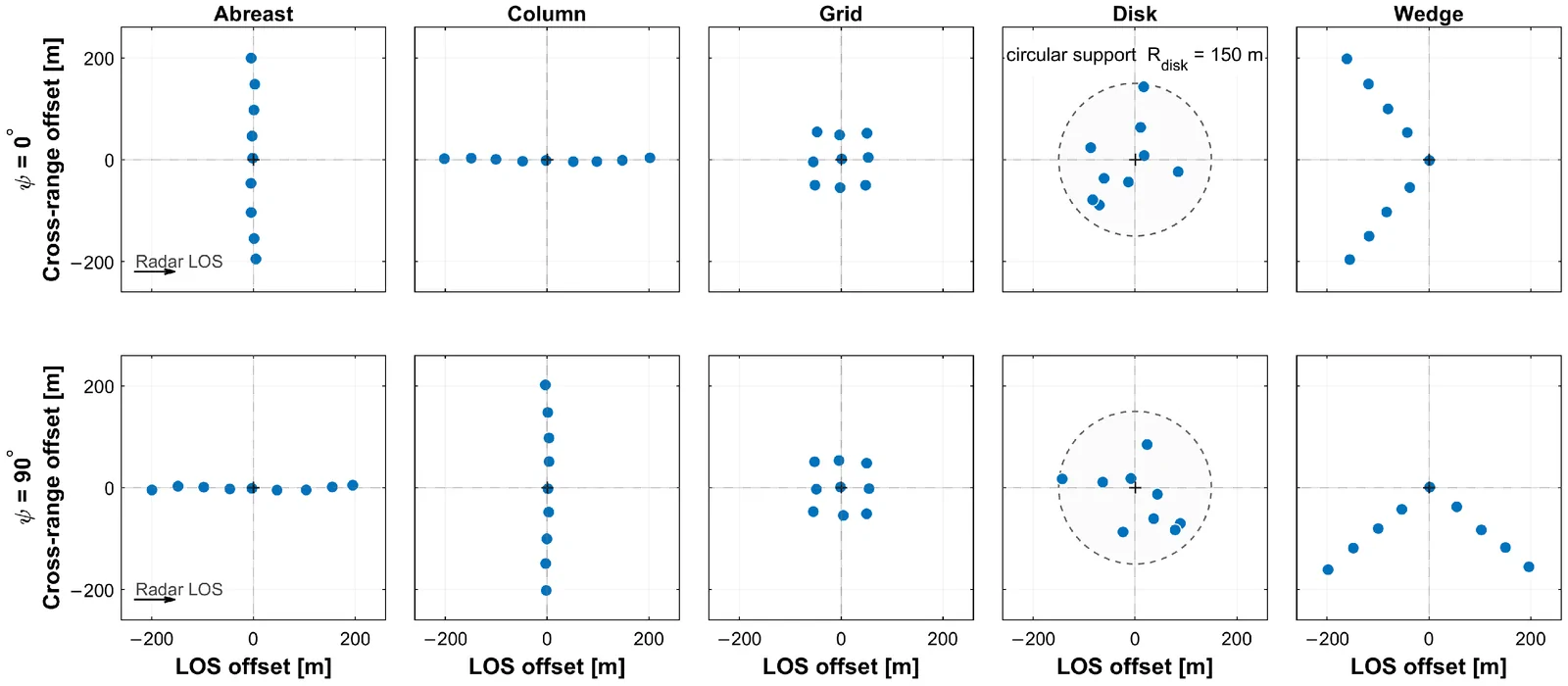
Representative swarm layouts in radar-centered coordinates for N=9 at a centroid range of $R_{c}=10\;\mathrm{km}$. The abreast, column, grid, and wedge formations use a nominal inter-member spacing of s=50m. The disk formation shows one representative random realization within a circular support of radius $R_{\mathrm{disk}}=150\;m$ while satisfying a minimum inter-target separation of $d_{\min}=15\;m$. The upper and lower rows correspond to $\psi =0^{\circ }$ and $\psi =90^{\circ }$, respectively. Positional jitter is omitted from the structured formations for clarity. Blue dots denote individual USVs, and the dashed circle indicates the circular support of the disk formation.

Representative formation geometries are shown in Figure 1. Stage 1 evaluates all five formations at the common reference cardinality $N_{\mathrm{ref}}=9$, which accommodates the prescribed 3×3 grid while remaining within the tested cardinality range. The cardinality-dependent comparison between Stage 1 and Stage 2 uses four representative geometries: the abreast formation at $\psi =0^{\circ }$ and $90^{\circ }$ as cases oriented predominantly in the transverse direction and along the RLOS, respectively, the disk formation at $\psi =0^{\circ }$ as a case distributed in two dimensions, and the abreast formation at $\psi =60^{\circ }$ as an oblique condition identified as group-observable in Stage 1. The disk is used instead of the grid for the cardinality dependent analysis because it can be generated consistently over the full tested cardinality range.

### 2.3. Target and Maritime Observation Model

For a given swarm geometry, the received radar data are represented over the array, range, and pulse dimensions. Let m=0,…,M−1, $q=0,\ldots ,N_{r}-1$, and $p=0,\ldots ,N_{p}-1$ denote the array element, range sample, and pulse indices, respectively. The received signal is modeled as

(7) $$x_{m,q,p}=\sum_{k=1}^{N}\sum_{\ell =1}^{N_{s}}x_{k,\ell }\left(m,q,p\right)+c_{m,q,p}+n_{m,q,p},$$

where *N* is the number of physical targets, $N_{s}$ is the number of scattering centers assigned to each target, $c_{m,q,p}$ denotes sea clutter, and $n_{m,q,p}$ denotes additive complex Gaussian noise.

The echo from the *ℓ*th scattering center of the *k*th target is modeled as

(8) $$x_{k,\ell }\left(m,q,p\right)=\alpha_{k,\ell }a_{m}\left(\theta_{k}\right)g\;\left(r_{q}-R_{k,\ell ,p}\right)\exp\left[j\left(2\pi f_{D,k}pT_{\mathrm{PRI}}-\frac{4\pi }{\lambda }R_{k,\ell ,p}\right)\right],$$

where $r_{q}$ is the range associated with the *q*th range sample, $\alpha_{k,\ell }$ is the complex scattering coefficient, and $\theta_{k}$ is the target azimuth angle. The monostatic Doppler frequency is $f_{D,k}=2v_{k}/\lambda$, where $v_{k}$ denotes the radial velocity of the *k*th target. For the uniform linear array with element spacing d=λ/2, the steering response is $a_{m}\left(\theta_{k}\right)=\exp\;\left(j2\pi dm\sin\theta_{k}/\lambda \right)$. For each Monte Carlo realization, the member radial velocities are drawn independently from a Gaussian distribution with mean $v_{c}$ and standard deviation $\sigma_{v}$.

To represent finite range resolution and range bin straddling, the range response is modeled using a sinc shaped matched filter response [24],

(9) $$g\;\left(r_{q}-R_{k,\ell ,p}\right)=\mathrm{sinc}\left(\frac{r_{q}-R_{k,\ell ,p}}{\Delta R}\right),$$

where sinc(x)=sin(πx)/(πx) and ΔR=c/(2B). This model allows changes in bandwidth to alter the range structure resolved from the same physical scene.

In the observability experiments, each physical target is represented by $N_{s}=3$ scattering centers distributed over a nominal hull length of $L_{h}=10\;m$. Their normalized longitudinal offsets are $\eta_{\ell }\in \left\{-0.45,0,0.45\right\}$. Each individual USV is assumed to keep its longitudinal axis oriented toward the radar, so that the longitudinal scattering center offsets lie along the local radar line of sight. The formation aspect rotation is applied only to the positions of the member centers about the swarm centroid. Changing ψ changes the projected arrangement of the member centers without changing the aspect of the individual targets. Under this assumption, the instantaneous range of the *ℓ*th scattering center is modeled as

(10) $$R_{k,\ell ,p}=R_{k}+\eta_{\ell }L_{h}+A_{r}\sin\left(2\pi f_{w}pT_{\mathrm{PRI}}+\phi_{r,k}\right),$$

where $R_{k}$ is the nominal range of the *k*th target at the current observation, $A_{r}$ is the amplitude of the wave-induced range perturbation, $f_{w}=1/T_{w}$, and $\phi_{r,k}$ is an independently generated initial phase. The baseline simulation uses $A_{r}=1$ m and $T_{w}=8$ s. The three-scatterer configuration is adopted as a compact nominal representation of distributed scattering along the vessel hull rather than as a unique physical scattering model. The sensitivity of the reported group observability and cardinality recovery results to the number and longitudinal extent of the scattering centers is examined in Section 4.4.

A controlled RCS normalization is applied to separate geometry-dependent cardinality information from the direct increase in nominal target strength that would occur if every additional swarm member were assigned the same RCS. In the Stage 1 analysis, $H_{E}$ denotes the hypothesis for a single extended target, and $H_{G}$ denotes the hypothesis for a target group containing *N* members. Under $H_{E}$, the single target uses the same hull model as each swarm member, with $N_{s}=3$ scattering centers over $L_{h}=10\;m$. A nominal reference RCS of $\sigma_{0}=0.1\;m^{2}$ is assigned to the reference target. Only relative RCS scaling enters the signal amplitude model, while the SNR of the reference target is controlled separately. The RCS values used under the two hypotheses are

(11) $$\sigma_{E}=\sigma_{0},\;\sigma_{G,k}=\frac{\sigma_{0}}{N},\;k=1,\ldots ,N.$$

Consequently, the nominal amplitude scale of each group member relative to the reference target satisfies

(12) $$\frac{A_{G,k}}{A_{E}}=\sqrt{\frac{\sigma_{G,k}}{\sigma_{E}}}=\frac{1}{\sqrt{N}}.$$

The normalization satisfies $\sum_{k=1}^{N}\sigma_{G,k}=\sigma_{0}$ for every tested cardinality. Its purpose is not to imply that a real target group has a cardinality-independent physical RCS, but to prevent a deterministic increase in nominal aggregate RCS from serving as a direct cardinality cue. This normalization fixes only the nominal aggregate RCS and does not force the instantaneous received power to be identical across realizations, since coherent superposition, scattering phases, and target fluctuations remain scene dependent. It removes a direct nominal amplitude cue while preserving cardinality dependent structure in the range–angle–Doppler measurements. To assess the sensitivity to this controlled normalization, a complementary condition in which the nominal RCS of each individual USV is held constant, $\sigma_{G,k}=\sigma_{0}$, is examined in Section 4.4, under which the aggregate nominal RCS increases naturally as $N\sigma_{0}$.

The temporal fluctuation of small vessel reflectivity is also included. Measurements from the Council for Scientific and Industrial Research (CSIR) Fynmeet trials showed that the reflectivity of small boats can be strongly affected by the surrounding sea surface and that its temporal behavior is correlated with the waves [25,26]. In the wave fluctuation model used here, the member-level complex amplitude follows a zero-mean circular complex Gaussian distribution with unit marginal power before RCS scaling. Its power is divided equally among the $N_{s}$ scattering centers, which are assigned independent random phases. Across repeated observations, the member-level complex amplitude is generated using the correlation model

(13) $$\rho_{A}\left(\Delta t\right)=\exp\left[-{\left(\frac{\Delta t}{\tau_{d}}\right)}^{2}\right]\cos\left(\frac{2\pi \Delta t}{T_{w}}\right),$$

where $T_{w}$ determines the wave period component and $\tau_{d}$ controls the decay of temporal correlation. The baseline value of $\tau_{d}$ is 6s. Because each classification decision in the present experiments uses a single observation, the temporal correlation in (13) does not affect the reported results and becomes relevant only when repeated observations are considered.

The maritime background is modeled using a compound-Gaussian model for K-distributed sea clutter. This model represents the clutter return as the product of a slowly varying texture component and a correlated complex Gaussian speckle component and is widely used for high-resolution maritime clutter [11,27,28,29]. The clutter contribution is generated as

(14) $$c_{m,q,p}=\sqrt{\frac{P_{c}}{N_{\theta }}}\sum_{j=1}^{N_{\theta }}a_{m}\left(\theta_{j}\right)\sqrt{\tau_{q,j}}\;u_{q,j}\left[p\right],$$

where $N_{\theta }$ denotes the number of angular clutter components, $\tau_{q,j}$ is the texture component, $u_{q,j}\left[p\right]$ is the pulse correlated complex Gaussian speckle, and $P_{c}$ denotes the clutter power. The clutter angles $\theta_{j}$ uniformly span $\left[-5^{\circ },5^{\circ }\right]$ with $N_{\theta }=21$.

The texture is independently generated across RA components according to

(15) $$\tau_{q,j}∼\mathrm{Gamma}\left(\nu ,\frac{1}{\nu }\right),\;E\left[\tau_{q,j}\right]=1.$$

The parameter ν controls the degree of non-Gaussian impulsiveness of the clutter, with smaller values producing heavier tails and more pronounced spiky behavior. The baseline value is set to ν=1.18, corresponding to an empirical shape parameter reported for an X-band Fynmeet measurement under low sea state conditions [26]. In addition to these marginal clutter statistics, temporal correlation of the speckle component is incorporated across pulses. Specifically, the pulse-to-pulse speckle correlation is modeled using a Gaussian Doppler spectrum [30], resulting in the temporal autocorrelation

(16) $$\rho_{c}\left[\ell \right]=\exp\left[-2{\left(\pi \sigma_{f}\ell T_{\mathrm{PRI}}\right)}^{2}\right]\exp\left(j2\pi f_{D,c}\ell T_{\mathrm{PRI}}\right),$$

where $f_{D,c}$ is the mean clutter Doppler frequency, and

(17) $$\sigma_{f}=\frac{1}{2\pi \tau_{c}}.$$

The baseline speckle correlation time is set to $\tau_{c}=13\;\mathrm{ms}$. This value lies within the approximately 10–20ms range of decorrelation times reported from the X-band Fynmeet measurements, where the temporal clutter characteristics were also found to depend on environmental conditions [26]. The mean clutter radial velocity is set to zero in the controlled simulations, giving $f_{D,c}=0$. The sensitivity to the texture shape parameter ν and the speckle correlation time $\tau_{c}$ is examined separately in Section 4.4.

With the clutter component specified, the remaining background term in (7) is additive complex Gaussian noise, modeled as

(18) $$n_{m,q,p}∼\mathrm{CN}\left(0,\sigma_{n}^{2}\right).$$

The target and clutter power levels are defined relative to this noise reference. The reference target SNR is specified per array element and per pulse before coherent processing and is set to 3dB, while the CNR is set to 11dB. Unlike ν and $\tau_{c}$, these values are not derived from measurements. They are fixed baseline operating points held constant across formation, aspect, and sensing resource conditions.

## 3. Observability Framework and Evaluation Protocol

The present analysis considers two related but distinct levels of observability. The first concerns whether a group of closely spaced targets can be distinguished from a single extended target. The second concerns whether the physical number of targets can be recovered once a group return is present.

### 3.1. Group Observability

Stage 1 evaluates whether the radar measurement contains sufficient structure to distinguish a target group from a single extended target. The two hypotheses are denoted by $H_{E}$ and $H_{G}$, representing the single extended target and group target hypotheses, respectively. The primary formation–aspect analysis evaluates the group hypothesis at the reference cardinality $N_{\mathrm{ref}}=9$. Group observability is additionally evaluated over N∈{3,…,12} for four representative geometries to examine its dependence on physical cardinality under the baseline sensing resources.

For a given formation *F*, viewing aspect ψ, and physical group cardinality *N*, group observability is quantified by

(19) $$P_{G}\left(F,\psi ,N\right)=P\left({\hat{H}}_{F,\psi ,N}=H_{G}|H_{G},F,\psi ,N\right),$$

where ${\hat{H}}_{F,\psi ,N}$ denotes the decision of the Stage 1 classifier constructed for the corresponding formation, aspect, and group cardinality. The corresponding false alarm probability for declaring the group hypothesis is

(20) $$P_{\mathrm{FA},G}\left(F,\psi ,N\right)=P\left({\hat{H}}_{F,\psi ,N}=H_{G}|H_{E}\right).$$

Under $H_{E}$, the physical scene contains a single extended target; the dependence of $P_{\mathrm{FA},G}$ on *F*, ψ, and *N* arises from the Stage 1 classifier constructed for the corresponding group condition.

The operating threshold is selected according to a Neyman–Pearson criterion [31], because Stage 1 imposes an explicit constraint on the group false alarm probability. The training procedure is described in Section 3.3. A combination of formation, aspect, and cardinality is regarded as group-observable when the 95% lower confidence bound (LCB) satisfies

(21) $${\mathrm{LCB}}_{95}\left[P_{G}\left(F,\psi ,N\right)\right]\geq \gamma_{G}$$

and the corresponding 95% upper confidence bound (UCB) satisfies

(22) $${\mathrm{UCB}}_{95}\left[P_{\mathrm{FA},G}\left(F,\psi ,N\right)\right]\leq \alpha_{G},$$

where $\gamma_{G}=0.90$ and $\alpha_{G}=0.05$ are used throughout the analysis. These values are prescribed operational criteria rather than parameters fitted to the simulated data: $\gamma_{G}=0.90$ requires a high probability of correct group recognition, while $\alpha_{G}=0.05$ imposes a restrictive upper limit on group false alarms. Confidence bounds are used instead of empirical point estimates so that a condition is declared observable only when the required performance is supported by the finite number of Monte Carlo realizations. The sensitivity to these thresholds is examined in Section 4.4.

### 3.2. Cardinality Recovery

Stage 2 evaluates whether the physical cardinality can be recovered from measurements generated under the group hypothesis. The tested cardinality set is

(23) N={3,4,…,12}.

For a given formation *F*, viewing aspect ψ, physical cardinality *N*, bandwidth *B*, and number of coherently processed pulses $N_{p}$, the probability of exact cardinality recovery is defined as

(24) $$P_{N}\left(F,\psi ,N;B,N_{p}\right)=P\left(\hat{N}=N|H_{G},F,\psi ,N,B,N_{p}\right),$$

where $\hat{N}$ is the estimated number of physical targets. Unlike Stage 1, which performs binary classification between $H_{E}$ and $H_{G}$, Stage 2 performs multiclass classification among the tested physical cardinalities using only observations generated under $H_{G}$.

The overall exact recovery rate is reported as the average over the tested cardinalities,

(25) $$P_{\mathrm{exact}}=\frac{1}{\left|N\right|}\sum_{N\in N}P_{N}\left(F,\psi ,N;B,N_{p}\right).$$

To assess whether coarse cardinality information remains available when exact recovery fails, the mean absolute cardinality error and the probability of an estimation error no larger than one target are considered:

(26) $${\mathrm{MAE}}_{N}=E\left[\left|\hat{N}-N\right|\right],$$

and

(27) $$P_{\mathrm{err}\leq 1}=P\left(\left|\hat{N}-N\right|\leq 1\right).$$

These metrics distinguish a complete loss of cardinality information from cases in which the measurement retains an ordinal or coarse indication of group size even though the exact integer value cannot be recovered. Recovery of a particular cardinality is regarded as certified only when

(28) $${\mathrm{LCB}}_{95}\left[P_{N}\left(F,\psi ,N;B,N_{p}\right)\right]\geq \gamma_{N},$$

where $\gamma_{N}=0.90$. The same 0.90 performance level is used for exact cardinality recovery so that the two stages are assessed against a consistent high-reliability criterion. This criterion is evaluated separately for each physical cardinality because the average recovery rate $P_{\mathrm{exact}}$ can remain high even when one or more cardinality classes are recovered poorly.

The two stages together define the cardinality concealment condition considered in this study. At a specified formation, viewing aspect, and physical cardinality under the baseline sensing resources, cardinality concealment occurs when group observability is certified but exact cardinality recovery is not certified. This condition is expressed as

(29) $$\left\{\begin{array}{cc} {\mathrm{LCB}}_{95}\left[P_{G}\left(F,\psi ,N\right)\right] & \geq \gamma_{G},\\ {\mathrm{UCB}}_{95}\left[P_{\mathrm{FA},G}\left(F,\psi ,N\right)\right] & \leq \alpha_{G},\\ {\mathrm{LCB}}_{95}\left[P_{N}\left(F,\psi ,N;B_{0},N_{p,0}\right)\right] & <\gamma_{N}. \end{array}\right.$$

The third condition denotes failure to certify that the exact recovery probability reaches $\gamma_{N}$ at the stated confidence level and does not establish that the underlying probability is below $\gamma_{N}$. Under the baseline sensing resources, the joint concealment condition is evaluated over N∈{3,…,12} for four representative geometries. The broader resource analysis evaluates the Stage 2 recovery criterion separately because Stage 1 group observability is not re-evaluated after the sensing resources are changed.

### 3.3. Classifier and Evaluation Protocol

The evaluation pipeline consists of detection, mapping to a common representation, feature extraction, and classification, which are described in that order. After Doppler processing, OS-CFAR [32] is applied along the range dimension to a post-Doppler detection statistic obtained by summing power over the array channels and retaining the maximum over Doppler bins. The CFAR settings are summarized in Table 1. Because the clutter and noise statistics of this detection depend on the sensing resource setting, the threshold multiplier is calibrated separately for each setting using target-free clutter and noise observations so that the prescribed false alarm probability is maintained. Stage 1 uses the baseline multiplier of 2.0947 for all formation–aspect conditions. In Stage 2, the multipliers for $2B_{0}$, $5B_{0}$, $10B_{0}$, $2N_{p,0}$, and $4N_{p,0}$ are 2.1549, 2.1268, 2.0994, 1.1916, and 1.1877, respectively, and each is held fixed across all geometries at that setting. If no range cell passes the threshold, the range cell with the largest detection statistic is retained as a fallback and no observation is discarded. Adjacent detections separated by no more than two native range bins are merged, and the detection with the largest statistic is retained as the representative range cell. For each observation, the common range support is centered on the representative range cell with the largest detection statistic. Across the resource sweep, the CFAR training cells, guard cells, order statistic, and detection merging rule are held fixed in native range cells, so their physical extent varies with the native range sampling interval. The physical representation support, common RA and RD grid sizes, PCA dimensionality, and regularized linear discriminant analysis (LDA) shrinkage parameter are held fixed.

To maintain a common feature definition across sensing resource settings, the measurements are mapped onto the fixed physical supports and common grids summarized in Table 1. The common grid is held fixed so that changes in radar resources do not alter the classifier input dimension. At the largest tested resource settings, the native resolution can be finer than the sampling interval of the common grid. In particular, the native range resolution at $10B_{0}$ is 1.5m, whereas the common range sampling interval is approximately 9.22m. Similarly, the native velocity resolution at $4N_{p,0}$ is approximately 0.13m/s, whereas the common Doppler sampling interval is approximately 0.525m/s. The sensitivity of the reported trends to this common grid representation is examined separately in Section 4.4.

For both representations, retained native range cells are assigned to the fixed common range intervals without range interpolation, and intervals containing no retained cell remain zero. The RA representation is formed from Bartlett spatial power [33], averaged over pulses and summed within each common range interval, after which the dominant angular response is centered, and the angular dimension is linearly interpolated onto the common angular grid. The RD representation is formed by applying a fast Fourier transform along the pulse dimension, with the resulting power averaged over the array channels and summed within each common range interval, then linearly interpolated onto the common velocity grid with samples outside the available native velocity support set to zero, and the dominant Doppler response subsequently centered.

Both observability stages use the same aligned RA and RD structural representation. Each representation is normalized by its total power before dimensionality reduction. For either representation *X*,

(30) $$\tilde{X}=\frac{X}{\sum_{i,j}X_{i,j}}.$$

This normalization suppresses direct dependence on the overall received amplitude while preserving the relative spatial and spectral structure within the measurement. PCA [34] is then performed separately for the RA and RD representations using only the training observations in each cross-validation fold. The leading 32 components from each representation are retained and concatenated to form the common feature vector

(31) $$z={\left[\begin{array}{cc} z_{\mathrm{RA}}^{T} & z_{\mathrm{RD}}^{T} \end{array}\right]}^{T}\in R^{64}.$$

The value of 32 components per representation is adopted as a fixed nominal dimensionality, keeping the resulting 64-dimensional feature vector small relative to the number of training observations in each fold (640 in Stage 1 and 960 in Stage 2). On the training data, this setting retains approximately 79–98% and 77–99% of the variance in the RA and RD representations, respectively. Its sensitivity is examined using 16 and 64 components in Section 4.4. LDA is applied with the same model form and shrinkage setting across all conditions to avoid introducing condition-dependent classifier hyperparameters.

In Stage 1, the feature vector z is used to discriminate between $H_{E}$ and $H_{G}$. For each training fold, the corresponding class means are denoted by $\mu_{E}$ and $\mu_{G}$, respectively. The pooled within-class covariance matrix is regularized using shrinkage [35],

(32) $$\tilde{S}=\left(1-\lambda_{\mathrm{sh}}\right)S+\lambda_{\mathrm{sh}}\frac{\mathrm{tr}(S)}{d}I,$$

where d=64 is the feature dimension. The value $\lambda_{\mathrm{sh}}=0.4$ is adopted as a fixed nominal setting rather than being tuned separately for individual geometry or resource conditions, and its sensitivity is examined in Section 4.4. The resulting linear discriminant direction is

(33) $$w={\tilde{S}}^{-1}\left(\mu_{G}-\mu_{E}\right),$$

and the corresponding discriminant score is

(34) $$s\left(z\right)={\left[z-\frac{\mu_{G}+\mu_{E}}{2}\right]}^{T}w.$$

In Stage 1, the Neyman–Pearson threshold [31] is selected from the $H_{E}$ training scores so that the one-sided 95% exact binomial upper bound on the false alarm probability does not exceed $\alpha_{G}=0.05$, and the selected threshold is applied to the corresponding test fold. The resulting held-out predictions are then pooled across folds to compute the final Stage 1 certification statistics.

In Stage 2, the same common feature vector z is used to discriminate among the physical cardinality classes n∈N. For each cardinality *n*, the mean of the training features is denoted by $\mu_{n}$. A common covariance matrix is estimated from the within-class deviations over all cardinality classes and regularized using (32). The distance between a test feature z and the class associated with cardinality *n* is

(35) $$d_{n}\left(z\right)={\left(z-\mu_{n}\right)}^{T}{\tilde{S}}^{-1}\left(z-\mu_{n}\right).$$

The estimated physical cardinality is then obtained as

(36) $$\hat{N}=\arg\min_{n\in N}d_{n}\left(z\right).$$

Under a Gaussian model in which the cardinality classes share a common covariance matrix and have equal prior probabilities, this decision rule corresponds to multiclass linear discriminant analysis [36]. The same covariance shrinkage parameter is used for both observability stages and is held fixed across geometry and resource conditions so that classifier adaptation does not become an additional source of performance variation.

All reported performance is obtained using stratified five-fold cross-validation [36]. No separate validation set is used because the classifier settings in the main analysis are fixed before evaluation rather than selected from the held-out data. Within each fold, PCA, class means, covariance estimates, and the Stage 1 decision threshold are determined using only the training observations, while the held-out fold is used exclusively for testing. Each Monte Carlo realization is assigned to exactly one fold and therefore does not appear in both the training and test partitions of the same evaluation. Predictions from the five held-out folds are pooled to compute the reported performance metrics.

Stage 1 uses 400 Monte Carlo realizations per hypothesis for each tested geometry and cardinality condition, while Stage 2 uses 120 realizations for each physical cardinality and resource condition. The reported Stage 1 lower and upper certification bounds and the Stage 2 bounds for each cardinality are one-sided 95% Clopper–Pearson exact binomial bounds [37] computed from the same pooled predictions. These bounds are used as operational certification criteria and are not simultaneous familywise confidence statements across all tested conditions. Paired differences between matched conditions are reported with 95% percentile bootstrap intervals obtained from 5000 resamples of the trial indices [38].

The number of representative range detections after merging adjacent CFAR detections, denoted by $n_{\mathrm{CFAR}}$, is used only as an auxiliary diagnostic. Its association with the true cardinality is monitored because detection count could provide a simpler counting cue than the distributed range–angle–Doppler structure. Additional analyses compare the primary representation with $n_{\mathrm{CFAR}}$ alone and with a representation from which its linear association with $n_{\mathrm{CFAR}}$ has been removed.

## 4. Validation of the Evaluation Pipeline

Before presenting the main observability results, this section examines whether the reported trends depend strongly on the measurement representation, alignment, geometry implementation, detection and representation choices, target and clutter modeling assumptions, classifier settings, and certification criteria. Unless otherwise stated, the checks use the constant aggregate RCS condition and the estimation procedure restricted to the training data defined in Section 3.

### 4.1. Canonical Processing Path

The effect of measurement representation was examined using the abreast formation at $\psi =60^{\circ }$. The two distributed representations retain the RA and RD responses but differ in their range cell selection. Two compressed representations were also considered. The sparse Bayesian learning (SBL) peak count [39] tests whether the number of resolvable angular components alone retains the relevant group information, whereas the angular scalar representation retains only the root mean square angular width, symmetric 90% energy width, and normalized angular entropy. All four representations were evaluated using the same observations and cross-validation folds, the constant aggregate RCS condition, the fixed baseline CFAR threshold multiplier, and the Neyman–Pearson decision rule. The comparison is summarized in Table 2.

The two distributed representations differ by 0.003 in $P_{G}$, whereas the compressed representations fall below the certification criterion. The apparent group observability therefore depends on how the measurement is represented. Unresolved members do not necessarily produce one angular peak each, so descriptors that summarize the angular response by peak count or by a few scalar statistics discard the structural differences that distinguish a swarm from a single extended target. The canonical processing path accordingly retains the distributed RA and RD structure, with range cell selection by CFAR adopted for consistency with the preceding detection stage.

### 4.2. Alignment Sensitivity

The canonical representation is centered with respect to its dominant angular and Doppler responses before classification. To determine whether the measured group separation depends strongly on this operation, aligned and unaligned representations were compared for the abreast formation at $\psi =60^{\circ }$. Target bearing was randomized independently across trials, and both conditions used the same observations, range support, cross-validation folds, and classifier settings.

Both aligned and unaligned representations satisfy the group observability criterion, with $P_{G}=0.993$ and 0.988 and corresponding lower confidence bounds of 0.981 and 0.974. In both cases, $P_{FA,G}=0.030$ with an upper confidence bound of 0.048. The paired difference in $P_{G}$ is 0.005 with a 95% percentile bootstrap interval of [0.000, 0.013], so alignment is not the source of the observed group separation. It is retained to remove the dependence on absolute bearing and radial velocity, so that comparisons across formation and aspect reflect relative measurement structure.

### 4.3. Rotational Symmetry and Physical Consistency

A physical consistency check is provided by the abreast and column formations. Under the formation definitions used here, complementary viewing aspects produce equivalent radar-relative geometries according to

(37) $$\psi_{A}+\psi_{C}=90^{\circ }.$$

The comparison was performed with the final Stage 1 processing pipeline. Common random numbers were used within each equivalent pair so that clutter, target fluctuation, motion, and noise realizations were matched.

The paired comparisons are summarized in Table 3. All paired confidence intervals (CIs) contain zero, and the largest observed difference is 0.030. The results are consistent with the expected rotational symmetry of the radar-relative geometry.

### 4.4. Confound and Robustness Checks

The remaining checks examine potential confounds and sensitivity to processing choices, target and clutter modeling assumptions, classifier settings, and certification criteria.

For the abreast formation at $\psi =60^{\circ }$, the number of representative range detections $n_{\mathrm{CFAR}}$ alone gives $P_{G}=0.968$ with ${\mathrm{LCB}}_{95}\left[P_{G}\right]=0.949$ and $P_{\mathrm{FA},G}=0.010$ with ${\mathrm{UCB}}_{95}\left[P_{\mathrm{FA},G}\right]=0.023$, which satisfies the group observability criterion. Group discrimination at this geometry can therefore be achieved from range support information alone. The same criterion is satisfied by the RA + RD representation computed over the fixed physical window (Table 2), so the group separation does not arise from the CFAR thresholding step itself.

Table 4a compares the canonical representation, $n_{\mathrm{CFAR}}$ alone, and a representation from which the linear association with $n_{\mathrm{CFAR}}$ is removed within each training fold.

At $B_{0}$ and $2B_{0}$, the point estimates obtained from $n_{\mathrm{CFAR}}$ alone are comparable to those of the full RA+RD representation, indicating that detection count is a substantial cardinality cue at low bandwidth. The residualized representation nevertheless remains above the uniform chance level, showing that cardinality information is not represented solely by a linear dependence on $n_{\mathrm{CFAR}}$. None of the three representations exhibits a monotonic improvement with bandwidth.

The effect of CFAR range selection on Stage 2 recovery was examined separately for the abreast formation at $\psi =90^{\circ }$ under the baseline sensing resources. Replacing the range cells retained by CFAR with all native range cells within the same fixed physical support increases $P_{\mathrm{exact}}$ from 0.207 to 0.228, with a paired difference of 0.021 and a 95% bootstrap interval of [−0.005,0.047]. For N=9, the exact recovery probability increases from 0.067 to 0.158, but its lower confidence bound remains only 0.106. Thus, CFAR range selection can affect individual cardinality estimates, but it does not account for the overall low exact recovery observed in this geometry.

The common grid was checked separately by doubling the range or Doppler sampling density while leaving the underlying observations unchanged. For the abreast formation at $\psi =90^{\circ }$, the change in $P_{\mathrm{exact}}$ from $B_{0}$ to $10B_{0}$ is 0.014 with the nominal range grid and 0.015 with the refined grid. The corresponding change from $N_{p,0}$ to $4N_{p,0}$ is 0.036 and 0.031 for the nominal and refined Doppler grids, respectively. The qualitative resource dependence is preserved under the tested twofold grid refinement.

For the positional robustness check, independent zero-mean Gaussian perturbations with standard deviations of 2.5 or 5m were added to the member positions. Table 4b reports the result using 60 trials per cardinality with independent random and cross-validation seeds, so its zero-jitter values differ slightly from the main Stage 2 experiment. Across the tested perturbation levels, exact recovery remains low and the change from $B_{0}$ to $10B_{0}$ does not exhibit a consistent improvement, so the qualitative result is preserved under the tested positional perturbations.

The following sensitivity analyses use Monte Carlo realizations generated independently from those of the main experiments. Their nominal-setting estimates therefore differ from the main results, and comparisons are made within each sensitivity analysis.

The sensitivity to the assumed target scattering representation was also examined to determine whether the reported cardinality behavior depends on the nominal three-scatterer model. In addition to the nominal model with three scattering centers over a 10 m hull, the analysis considered two and five scattering centers over the same hull length and three-scatterer models with longitudinal extents of 6 and 14 m.

As summarized in Table 5, exact cardinality recovery remains uncertified for every tested target representation. Across the four representative geometries at $B_{0}$, the largest class-specific lower confidence bound is 0.513, remaining well below $\gamma_{N}=0.90$, and no certified recovery is obtained at $10B_{0}$ for the abreast formation at $\psi =90^{\circ }$. Stage 1 group observability also remains high, with nine of the ten tested representation–geometry combinations satisfying the complete certification criterion; the single exception marginally exceeds the false-alarm upper-bound requirement with ${\mathrm{UCB}}_{95}\left[P_{\mathrm{FA},G}\right]=0.051$.

The sensitivity to the RCS normalization was examined by comparing the nominal condition, in which the aggregate nominal RCS is held constant ($\sigma_{G,k}=\sigma_{0}/N$), with a complementary condition in which the nominal RCS of each individual USV is held constant ($\sigma_{G,k}=\sigma_{0}$).

As shown in Table 6, holding the RCS of each individual USV constant changes the quantitative recovery performance in some geometries, but no physical cardinality achieves certified exact recovery. Stage 1 group observability also remains certified for both tested abreast geometries under the fixed-member RCS condition, with $P_{G}=0.985$ and 0.990 for $\psi =60^{\circ }$ and $90^{\circ }$, respectively.

The sensitivity to the assumed sea clutter parameters was also examined by varying the texture shape parameter ν and the speckle correlation time $\tau_{c}$ separately around their baseline values. For each clutter condition, the CFAR multiplier was recalibrated using target-free clutter and noise observations to maintain the prescribed false-alarm operating point.

As summarized in Table 7, varying the texture shape parameter ν does not change the Stage 1 certification outcome for the two tested abreast geometries, and the same result is obtained for $\tau_{c}=10$ ms. In contrast, at $\tau_{c}=20$ ms, the group selection probabilities decrease to 0.850 and 0.887 for $\psi =60^{\circ }$ and $90^{\circ }$, respectively, and neither condition remains certified. Stage 2 exact cardinality recovery remains uncertified for every tested clutter condition, with the largest class-specific lower confidence bound equal to 0.513, well below $\gamma_{N}=0.90$. Thus, the exact-cardinality limitation is preserved across the tested clutter parameters, although Stage 1 group observability decreases for the longest tested correlation time.

The sensitivity to the PCA dimensionality was examined by retaining 16, 32, or 64 components from each of the RA and RD representations. PCA was fitted using only the training observations within each cross-validation fold, and the retained variance was evaluated from the corresponding training data. The covariance shrinkage parameter was also varied from 0.1 to 0.8 while keeping the PCA dimensionality fixed at 32 components per representation.

Table 8 summarizes the sensitivity to the PCA dimensionality and covariance shrinkage. The reported ranges correspond to the four representative geometries, and the retained variance is averaged over the cross-validation folds; for the shrinkage analysis, the PCA dimensionality is fixed at 32 components per representation. Across all tested settings, quantitative recovery varies to some extent, but exact cardinality recovery remains uncertified, with the largest ${\mathrm{LCB}}_{95}\left[P_{N}\right]$ equal to 0.504 for the PCA analysis and 0.513 for the shrinkage analysis. For Stage 1, both tested abreast geometries remain certified with 16 and 32 PCA components and over the tested shrinkage range, whereas 64 PCA components marginally exceed the false-alarm requirement.

The sensitivity to the certification criteria was also examined using $\alpha_{G}\in \left\{0.025,0.05,0.10\right\}$ and $\gamma_{G},\gamma_{N}\in \left\{0.85,0.90,0.95\right\}$, with the Neyman–Pearson threshold reselected for each $\alpha_{G}$. With $\gamma_{G}=0.90$, the number of certified formation–aspect conditions changes from 8 of 35 to 12 of 35 and 25 of 35 as $\alpha_{G}$ increases from 0.025 to 0.05 and 0.10, respectively. With $\alpha_{G}=0.05$, the corresponding numbers are 18, 12, and 10 of 35 for $\gamma_{G}=0.85$, 0.90, and 0.95. In contrast, no tested cardinality achieves certified exact recovery even at $\gamma_{N}=0.85$, because the largest ${\mathrm{LCB}}_{95}\left[P_{N}\right]$ over the four representative geometries and the tested resource settings remains approximately 0.52. Thus, the exact-cardinality limitation is preserved over the tested certification levels, whereas the extent of the certified Stage 1 observable region depends on the prescribed operating criteria.

Finally, the dependence of fragmentation on bandwidth was examined to distinguish resolution of different physical members from resolution of multiple scattering components within one member. The control case with a single target used the extended USV model with the RCS of each member in the N=9 case, i.e., $\sigma_{0}/9$. The probability that a single physical USV produces multiple associated detections increases from 0.013 at $B_{0}$ to 0.537 at $10B_{0}$, while its mean detected range span increases from 0.28 m to 6.15 m. At the swarm level, the resolved member fraction for the abreast formation at $\psi =90^{\circ }$ initially increases from 0.673 at $B_{0}$ to 0.733 at $2B_{0}$, but decreases to 0.639 at $10B_{0}$, while the mean number of associated detections per resolved member increases from approximately 1.01 at $B_{0}$ to 1.07 at $2B_{0}$ and 2.75 at $10B_{0}$. The $\psi =60^{\circ }$ case exhibits the same qualitative behavior.

At moderate bandwidth the additional resolution primarily separates different members, whereas at larger bandwidths it increasingly resolves scattering components within individual members, so the number of detections no longer tracks the number of physical targets.

## 5. Experimental Results

### 5.1. Formation–Aspect Dependence of Group Observability

**Figure 2:**
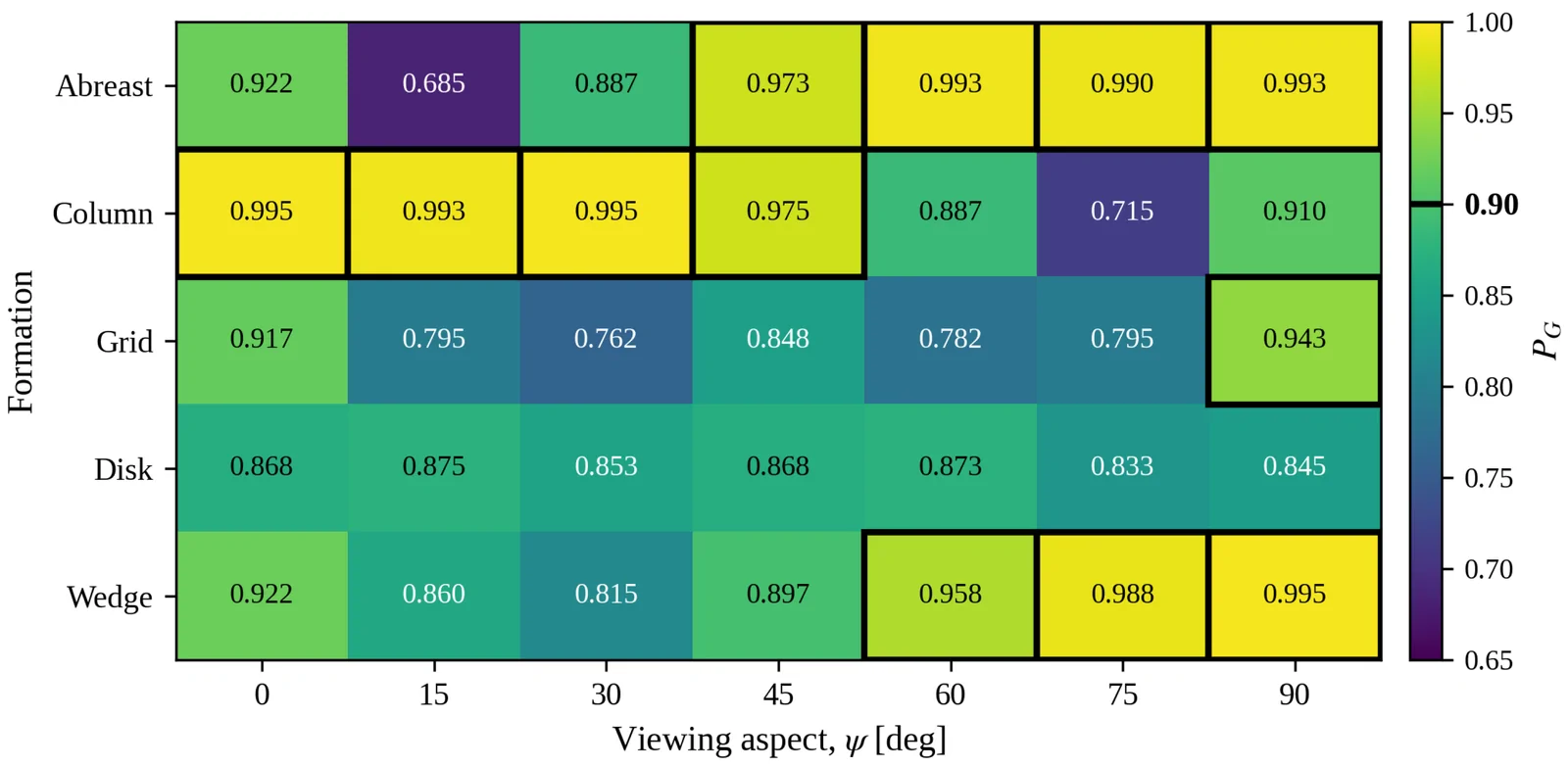
Group observability over the tested formation–aspect conditions at $N_{\mathrm{ref}}=9$. Color indicates the empirical group selection probability $P_{G}$. Boxed cells identify conditions satisfying both ${\mathrm{LCB}}_{95}\left[P_{G}\right]\geq 0.90$ and ${\mathrm{UCB}}_{95}\left[P_{\mathrm{FA},G}\right]\leq 0.05$. The displayed values and certification decisions are the directly estimated results and are not symmetrized across geometrically equivalent conditions.

Stage 1 was evaluated for five formation types and seven viewing aspects, giving 35 formation–aspect conditions. Among these conditions, 12 satisfy both group observability certification requirements. Figure 2 shows that group observability varies by more than 0.3 in $P_{G}$ across the tested conditions and that the certified conditions cluster at opposite aspect ranges for the two linear formations. The abreast formation is certified from $\psi =45^{\circ }$ to $90^{\circ }$, whereas the column formation is certified from $\psi =0^{\circ }$ to $45^{\circ }$, consistent with the rotational equivalence examined in Section 4.3. The wedge formation is certified from $\psi =60^{\circ }$ to $90^{\circ }$, and none of the tested disk conditions is certified. The square grid illustrates the distinction between physical symmetry and certification from a finite sample: it is geometrically equivalent at $\psi =0^{\circ }$ and $90^{\circ }$ under a $90^{\circ }$ rotation, but the empirical values of 0.917 and 0.943 fall on opposite sides of the certification boundary. The intermediate aspects show the expected approximate symmetry, with identical results at $15^{\circ }$ and $75^{\circ }$ and similar results at $30^{\circ }$ and $60^{\circ }$. The abreast formation at $\psi =0^{\circ }$ gives $P_{G}=0.922$ but is not certified, since its lower confidence bound is 0.897, and the upper bound on its group false alarm probability is 0.057.

### 5.2. Divergence Between Group and Cardinality Observability

At $N=N_{\mathrm{ref}}=9$ and the baseline sensing resources, we compare the certification outcomes for the abreast configurations at $\psi =60^{\circ }$ and $90^{\circ }$. For both geometries, Stage 1 gives $P_{G}=0.993$ with ${\mathrm{LCB}}_{95}\left[P_{G}\right]=0.981$ and $P_{\mathrm{FA},G}=0.030$ with ${\mathrm{UCB}}_{95}\left[P_{\mathrm{FA},G}\right]=0.048$, so both satisfy the group observability criterion. Stage 2 gives $P_{9}=0.108$ and 0.067, respectively, with lower confidence bounds of 0.065 and 0.034, so neither satisfies the exact recovery criterion.

The two probabilities correspond to different inference tasks and are not directly comparable as classification accuracies. The relevant comparison is between their certification outcomes: group observability is certified at both geometries, whereas exact cardinality recovery at N=9 is not. These two baseline conditions therefore satisfy the cardinality concealment definition in (29). The condition occurs both for a formation whose projected extent lies predominantly along the RLOS and for an oblique geometry.

**Figure 3:**
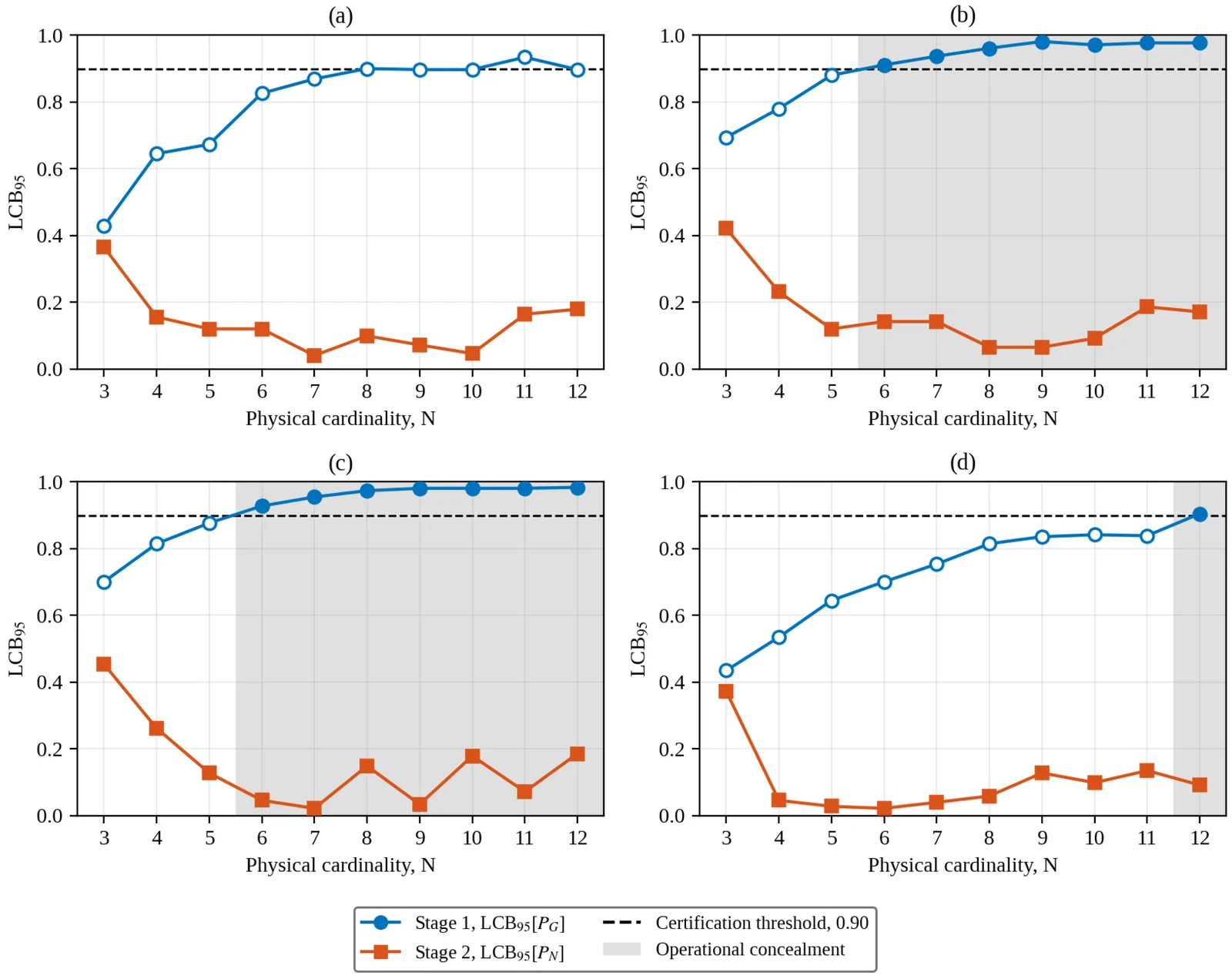
Cardinality-dependent divergence between group observability and exact cardinality recovery under the baseline sensing resources. (**a**) Abreast formation at $\psi =0^{\circ }$. (**b**) Abreast formation at $\psi =60^{\circ }$. (**c**) Abreast formation at $\psi =90^{\circ }$. (**d**) Disk formation at $\psi =0^{\circ }$. The blue curve shows the one-sided 95% lower confidence bound ${\mathrm{LCB}}_{95}\left[P_{G}\right]$ for Stage 1 group observability, and the orange curve shows ${\mathrm{LCB}}_{95}\left[P_{N}\right]$ for Stage 2 exact cardinality recovery. The dashed line denotes the common certification threshold of 0.90. Filled blue markers indicate cardinalities that satisfy the full Stage 1 certification criterion, including the group false alarm requirement. Gray shaded regions indicate the tested cardinalities for which Stage 1 group observability is certified while Stage 2 exact cardinality recovery remains uncertified.

Figure 3 extends this comparison over N∈{3,…,12} for the four representative geometries. Stage 1 is evaluated at each cardinality with the same observation model and evaluation protocol as in Figure 2, and the N=9 values are taken directly from that evaluation. Across the four geometries, ${\mathrm{LCB}}_{95}\left[P_{G}\right]$ shows an overall increasing tendency with physical cardinality, although the trend is not strictly monotonic. Because the aggregate nominal RCS is held constant across *N*, this tendency cannot be attributed to a deterministic increase in nominal aggregate return power. For the structured abreast formations, increasing *N* also increases the physical formation extent, whereas the disk retains a fixed spatial support and becomes denser as additional members are added. For the abreast formations at $\psi =60^{\circ }$ and $90^{\circ }$, group observability is certified for every tested cardinality from N=6 through N=12, whereas ${\mathrm{LCB}}_{95}\left[P_{N}\right]$ does not exceed 0.19 over the same range.

For N=3 through 5, group observability is not certified for any of the four representative geometries under the baseline sensing resources. The disk formation is certified only at N=12, with a lower confidence bound of 0.905, while the abreast formation at $\psi =0^{\circ }$ is not certified at any tested cardinality. At N=11 for the latter geometry, ${\mathrm{LCB}}_{95}\left[P_{G}\right]=0.934$ exceeds the threshold, but the upper bound on the group false alarm probability is 0.066, so the certification criterion is not satisfied.

Beyond exact recovery, the aggregate metrics $P_{\mathrm{exact}}$, ${\mathrm{MAE}}_{N}$, and $P_{\mathrm{err}\leq 1}$ computed over all ten tested cardinality classes indicate how much information about group size remains. For $\psi =60^{\circ }$, the baseline values are $P_{\mathrm{exact}}=0.222$, ${\mathrm{MAE}}_{N}=1.76$, and $P_{\mathrm{err}\leq 1}=0.522$. For $\psi =90^{\circ }$, the corresponding values are 0.207, 1.75, and 0.563. For reference, uniform guessing over the ten cardinality classes gives $P_{\mathrm{err}\leq 1}=0.28$ and ${\mathrm{MAE}}_{N}=3.3$. The estimates retain coarse information about group size, although no tested cardinality satisfies the exact recovery criterion at the baseline resource condition.

**Figure 4:**
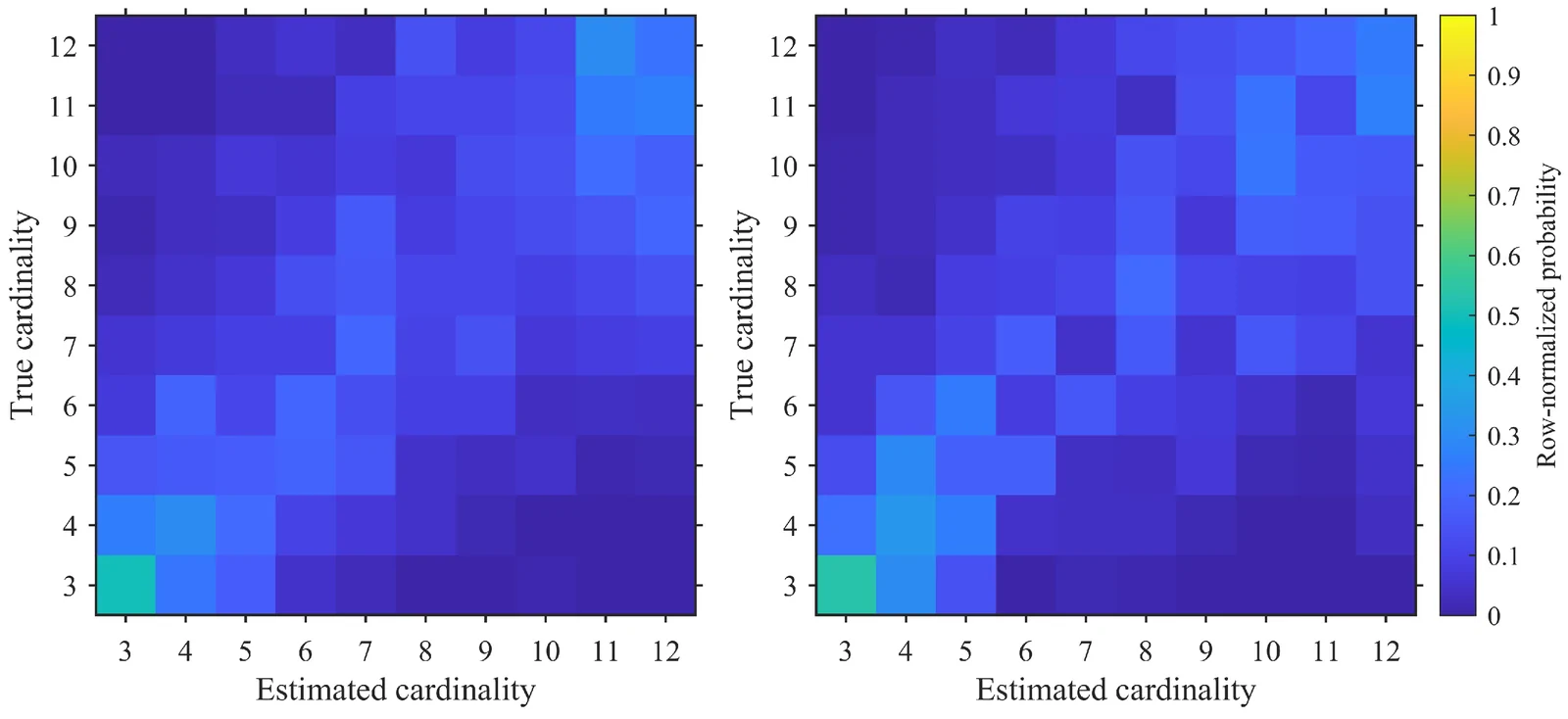
Confusion matrices normalized by row for physical cardinality recovery under the baseline sensing resources. (**a**) Abreast formation at $\psi =60^{\circ }$. (**b**) Abreast formation at $\psi =90^{\circ }$. Rows indicate the true physical cardinality and columns indicate the estimated cardinality. Each matrix is computed from the pooled out-of-fold predictions used for the reported baseline Stage 2 performance.

Figure 4 further shows the class specific structure of the baseline cardinality errors. For both viewing aspects, the row normalized probability is spread over several estimated cardinalities rather than concentrated along the main diagonal, indicating that the low exact recovery rate is not driven by only one poorly recovered cardinality class. At the same time, the dominant probability mass generally shifts toward larger estimated cardinalities as the true cardinality increases, leaving a broad diagonal trend across the matrices.

### 5.3. Stage 2 Resource Dependence of Cardinality Recovery

The Stage 2 resource analysis examines whether exact cardinality recovery improves as bandwidth or coherent dwell is increased. Four representative geometries are considered: abreast at $\psi =0^{\circ }$, disk at $\psi =0^{\circ }$, and abreast at $\psi =60^{\circ }$ and $90^{\circ }$.

**Figure 5:**
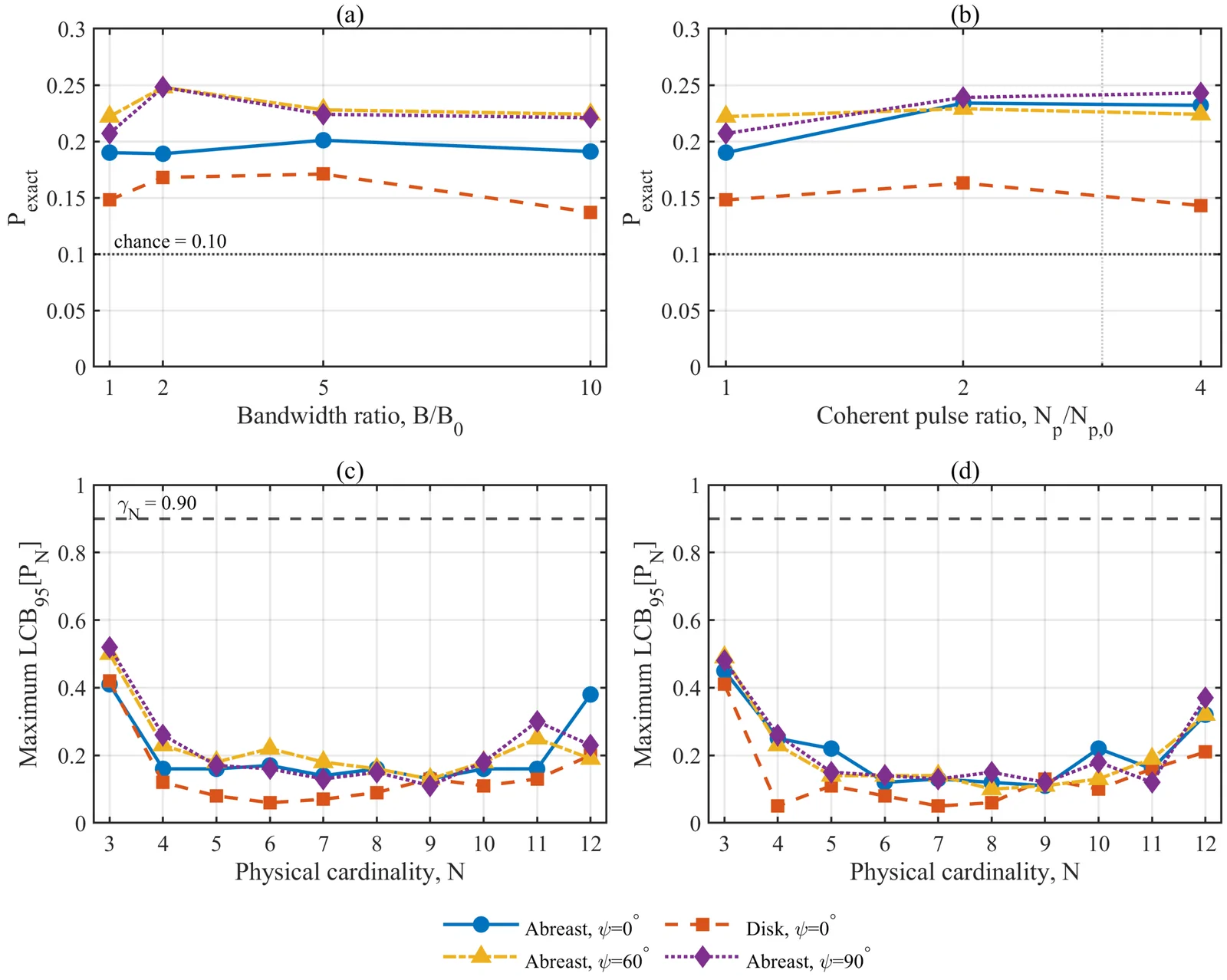
Cardinality recovery as a function of the tested sensing resources for four representative radar-relative geometries. (**a**) Overall exact recovery probability $P_{\mathrm{exact}}$ as bandwidth is increased from $B_{0}$ to $10B_{0}$. (**b**) Corresponding result as the number of coherently processed pulses is increased from $N_{p,0}$ to $4N_{p,0}$. The dotted line in (**a**,**b**) denotes the uniform chance level of 0.10. (**c**) For each physical cardinality *N*, the maximum one-sided 95% lower confidence bound of $P_{N}$ attained over the tested bandwidth settings. (**d**) Corresponding bound attained over the tested coherent pulse settings. The gray dashed line in (**c**,**d**) denotes the certification criterion $\gamma_{N}=0.90$.

Figure 5a shows only modest changes in overall exact recovery across the tested bandwidth settings. For the abreast configurations at $\psi =60^{\circ }$ and $90^{\circ }$, $P_{\mathrm{exact}}$ peaks at 0.248 at $2B_{0}$ and does not increase further at $5B_{0}$ or $10B_{0}$, so the bandwidth dependence is not monotonic. This trend is consistent with the intra-target fragmentation observed in Section 4.4. Figure 5b shows similarly limited changes with coherent dwell. Across the complete resource sweep, exact recovery remains between approximately 0.14 and 0.25, the mean absolute error does not fall below 1.54 targets, and $P_{\mathrm{err}\leq 1}$ does not exceed 0.585.

Figure 5c,d apply the certification rule for each cardinality, where the plotted value is the largest lower confidence bound attained over the tested settings on the corresponding resource axis. No tested physical cardinality achieves certified exact recovery for any of the four representative geometries at any tested bandwidth or coherent dwell setting. The largest lower confidence bound is approximately 0.52 over the bandwidth sweep and approximately 0.49 over the coherent dwell sweep, both far below $\gamma_{N}=0.90$. The group observability criterion was not re-evaluated at the increased resource settings, so the results in this subsection concern the cardinality recovery task alone.

## 6. Discussion

The divergence between group recognition and exact cardinality recovery appears over a range of the tested cardinalities, and the tested geometries show two distinct failure regimes. In the transverse abreast geometry, the members occupy a range extent smaller than one baseline range-resolution cell and are distributed primarily in cross-range. Member-to-member range separation is not resolved, so the group response differs from that of a single extended target chiefly through cross-range and Doppler structure. With the aggregate nominal RCS held constant, this structure is not sufficient for certification at any tested cardinality, and the limitation already appears at group recognition. The disk, whose fixed support becomes denser rather than larger as members are added, likewise fails the group observability criterion except at N=12.

The abreast geometries with substantial projection along the RLOS distribute their members over many range cells, and their projected range extent grows with cardinality. This range support is a strong group cue, strong enough that $n_{\mathrm{CFAR}}$ alone yields certification at $\psi =60^{\circ }$. It does not give a one-to-one correspondence between detections and physical members. At $\psi =90^{\circ }$ and the baseline bandwidth, only about two thirds of the physical members produce separately associated detections (Section 4.4). Replacing the CFAR-retained range cells with the full fixed physical window raises the recovery of some cardinalities, but the overall exact recovery stays low, and the N=9 condition is still uncertified. In these RLOS-projected abreast conditions, range-domain support can therefore be sufficient for group recognition without being sufficient for exact cardinality recovery. Combined with the transverse and disk cases, these results indicate that cardinality concealment depends jointly on physical cardinality and radar-relative projected geometry. For the two baseline abreast cases, the errors remain broadly distributed around the true cardinality even though exact recovery is uncertified, group recognition, coarse cardinality estimation, and certified exact cardinality recovery are distinct sensing outcomes in these experiments.

Additional sensing resources do not yield certified exact recovery within the tested resource envelope. As finer range resolution leads to increasing intra-target fragmentation (Section 4.4), detection count ceases to track physical cardinality, while longer coherent dwell likewise yields no certified exact recovery. For an MFR resource manager, the benefit of additional bandwidth or dwell for exact cardinality recovery is therefore limited, is not monotonic in bandwidth, and depends on the geometry and the processing chain.

All results are obtained at a single SNR–CNR operating point of 3 dB and 11 dB. Although the sensitivity to ν and $\tau_{c}$ is examined separately, the bandwidth sweep holds the clutter parameters fixed and therefore does not model resource-dependent changes in clutter power or statistics per resolution cell [11,28]. Stage 1 group observability is also sensitive to the speckle correlation time, with neither tested abreast geometry remaining certified at $\tau_{c}=20$ ms (Table 7). The single-target hypothesis is a 10m vessel and does not represent an extended object comparable in size to the swarm. CFAR range selection, the detection-merging rule fixed in native range cells, mapping to common grids that remain coarser than the finest native resolutions even after the tested twofold refinement, power normalization, dimensionality reduction, and the regularized linear discriminant can all limit how much of the native measurement structure reaches the decision, and a more expressive estimator could exploit structure not captured here. Each decision also uses a single observation. Conversely, each evaluation is conditioned on a specified formation and aspect, with Stage 1 additionally conditioned on group cardinality, which removes nuisance uncertainty that would be present under unknown geometry. Accordingly, the reported cardinality-recovery limitation pertains to the evaluated simulation model and signal-processing framework rather than to radar systems in general.

## 7. Conclusions

This study introduced a two-stage framework for evaluating group observability and exact cardinality recovery of maritime target swarms. At N=9, group observability is certified in 12 of the 35 tested formation–aspect conditions and depends strongly on the radar-relative projected geometry rather than on the nominal formation label alone. For the abreast formations at $\psi =60^{\circ }$ and $90^{\circ }$, $P_{G}=0.993$, whereas the corresponding exact cardinality recovery probabilities are 0.108 and 0.067. Under the baseline sensing resources, the concealment condition persists over N=6,…,12 for these two configurations. Coarse cardinality information nevertheless remains for these two configurations, with 52–56% of the estimates lying within one target of the true value, compared with 28% for uniform guessing. Across the four representative geometries, group observability is not certified for any tested condition at N=3,…,5. At larger cardinalities, two distinct failure regimes emerge: transverse and disk geometries are limited primarily at Stage 1, whereas the RLOS-projected abreast geometries are group-observable over N=6,…,12 while exact cardinality recovery remains uncertified.

For the two abreast geometries that exhibit concealment over N=6,…,12, the overall exact-recovery rate $P_{\mathrm{exact}}$, averaged over N=3,…,12, peaks at 0.248 at $2B_{0}$ and does not increase further with bandwidth, consistent with increasing intra-target fragmentation at larger bandwidths. Across all four representative geometries, all tested cardinalities, and all tested resource settings, the largest class-specific ${\mathrm{LCB}}_{95}\left[P_{N}\right]$ remains approximately 0.52. Increasing the tested sensing resources up to $10B_{0}$ and $4N_{p,0}$ therefore does not yield certified exact recovery for any tested cardinality. The lack of certified exact recovery is also preserved under the tested variations in target scattering representation, RCS normalization, clutter parameters, PCA dimensionality, covariance shrinkage, and certification criteria. Within the evaluated simulation model and signal-processing framework, these results show that successful group recognition does not necessarily imply reliable exact cardinality recovery, but they do not establish a general limitation of radar systems. Future work will examine more diverse observations, estimators, and unknown formation conditions to improve cardinality recovery.

## Figures and Tables

**Table 1:** Baseline radar, scenario, processing, and evaluation parameters. Sensing resources are varied relative to the baseline pair $\left(B_{0},N_{p,0}\right)$, while the remaining parameters are held fixed unless otherwise stated.

Group	Parameter	Value
Radar	Array elements *M*, spacing	64, λ/2
	Carrier frequency	9 GHz
	Baseline bandwidth $B_{0}$, range resolution $\Delta R_{0}$	10 MHz, 15 m
	PRF, $T_{\mathrm{PRI}}$	4 kHz, 0.25 ms
	Baseline coherent pulses $N_{p,0}$	128
	Azimuth scan range	$\pm 5^{\circ }$
Scenario	Swarm centroid range $R_{c}$	10 km
	Centroid velocity $v_{c}$, velocity spread $\sigma_{v}$	25 m/s, 0.3 m/s
	Reference and tested cardinalities	$N_{\mathrm{ref}}=9$, N=3,…,12
	Inter-member spacing *s*	50 m
	Disk radius $R_{\mathrm{disk}}$, minimum separation $d_{\min}$	150 m, 15 m
	Scattering centers $N_{s}$, hull length $L_{h}$	3, 10 m
	Reference target RCS $\sigma_{0}$	0.1 m^2^
Environment	Target SNR, CNR	3 dB, 11 dB
	Clutter shape ν, correlation time $\tau_{c}$	1.18, 13 ms
	Wave range amplitude $A_{r}$, period $T_{w}$	1 m, 8 s
	Amplitude decorrelation time $\tau_{d}$	6 s
Detection	CFAR detector	1-D OS-CFAR along range
	Training cells, guard cells	10, 3 per side
	Order statistic, target $P_{\mathrm{FA}}$	12th of 20, $10^{-3}$
	Baseline CFAR multiplier	2.0947
Representation	Common range and velocity supports	±590 m, [−33.33, 33.33]m/s
	Common range–angle (RA) and range–Doppler (RD) grids	128×32, 128×128
	Retained PCA components	32 RA, 32 RD
Evaluation	Cross-validation folds	5
	Stage 1/Stage 2 trials	400 per hypothesis/120 per class
	Group criteria $\gamma_{G}$, $\alpha_{G}$	0.90, 0.05
	Cardinality criterion $\gamma_{N}$	0.90
	Confidence bounds	One-sided 95% exact binomial
	Covariance shrinkage parameter $\lambda_{\mathrm{sh}}$	0.4

**Table 2:** Ablation of the measurement representation for the abreast formation at $\psi =60^{\circ }$. Group observability is certified when both ${\mathrm{LCB}}_{95}\left[P_{G}\right]\geq 0.90$ and ${\mathrm{UCB}}_{95}\left[P_{FA,G}\right]\leq 0.05$ are satisfied.

Representation	Range Cells	$P_{G}$ [LCB_95_]	$P_{\mathrm{FA},G}$ [UCB_95_]	Certified
RA + RD	CFAR retained	0.993 [0.981]	0.030 [0.048]	Yes
RA + RD	Fixed window	0.990 [0.977]	0.030 [0.048]	Yes
SBL peaks	CFAR retained	0.083 [0.061]	0.022 [0.039]	No
Angular scalars	CFAR retained	0.000 [0.000]	0.030 [0.048]	No

**Table 3:** Group observability for complementary abreast and column viewing aspects.

$\psi_{A}$	$\psi_{C}$	$P_{G}^{A}$	$P_{G}^{C}$	$\Delta P_{G}$ [95% CI]
$0^{\circ }$	$90^{\circ }$	0.922	0.910	0.012 [−0.020, 0.045]
$15^{\circ }$	$75^{\circ }$	0.685	0.715	−0.030 [−0.077, 0.018]
$30^{\circ }$	$60^{\circ }$	0.887	0.887	0.000 [−0.033, 0.033]
$45^{\circ }$	$45^{\circ }$	0.973	0.975	−0.002 [−0.018, 0.013]
$60^{\circ }$	$30^{\circ }$	0.993	0.995	−0.002 [−0.013, 0.005]
$75^{\circ }$	$15^{\circ }$	0.990	0.993	−0.003 [−0.013, 0.005]
$90^{\circ }$	$0^{\circ }$	0.993	0.995	−0.002 [−0.010, 0.005]

**Table 4:** Confound and robustness checks for Stage 2. (**a**) Exact cardinality recovery for the abreast formation at $\psi =90^{\circ }$ using the distributed representation, $n_{\mathrm{CFAR}}$ alone, and the linearly residualized representation. (**b**) Exact cardinality recovery under positional perturbations of the two certified abreast geometries.

**(a) Detection count diagnostic**			
**Resource**	**RA + RD**	$n_{\mathrm{CFAR}}$ ** Only**	**Residualized**
$B_{0}$	0.207	0.224	0.160
$2B_{0}$	0.248	0.251	0.185
$5B_{0}$	0.224	0.198	0.193
$10B_{0}$	0.221	0.190	0.183
**(b) Positional jitter robustness**			
ψ	Jitter std. [m]	$B_{0}$	$10B_{0}$
$60^{\circ }$	0.0	0.203	0.207
$60^{\circ }$	2.5	0.237	0.212
$60^{\circ }$	5.0	0.205	0.213
$90^{\circ }$	0.0	0.220	0.210
$90^{\circ }$	2.5	0.227	0.198
$90^{\circ }$	5.0	0.207	0.222

**Table 5:** Sensitivity to the target scattering-center representation. Stage 1 reports the number of certified conditions among the two abreast geometries at $\psi =60^{\circ }$ and $90^{\circ }$ for N=9. The $B_{0}$ column reports the maximum ${\mathrm{LCB}}_{95}\left[P_{N}\right]$ over the four representative geometries, whereas the $10B_{0}$ column corresponds to the abreast formation at $\psi =90^{\circ }$.

Model	Stage 1 Cert.	$B_{0}$ Max. LCB	$10B_{0}$ Max. LCB
$N_{s}=2,\;L_{h}=10$ m	2/2	0.430	0.454
$N_{s}=3,\;L_{h}=6$ m	2/2	0.438	0.373
$N_{s}=3,\;L_{h}=10$ m	2/2	0.504	0.397
$N_{s}=3,\;L_{h}=14$ m	2/2	0.479	0.463
$N_{s}=5,\;L_{h}=10$ m	1/2	0.513	0.421

**Table 6:** Sensitivity to the RCS normalization. The fixed-total condition uses $\sigma_{G,k}=\sigma_{0}/N$, whereas the fixed-member condition uses $\sigma_{G,k}=\sigma_{0}$. The maximum LCB denotes the largest ${\mathrm{LCB}}_{95}\left[P_{N}\right]$ over N∈{3,…,12} under the baseline sensing resources.

Geometry	Fixed Total		Fixed Member	
	$P_{\mathrm{exact}}$	Max. LCB	$P_{\mathrm{exact}}$	Max. LCB
Abreast, $\psi =0^{\circ }$	0.191	0.357	0.208	0.365
Disk, $\psi =0^{\circ }$	0.128	0.254	0.166	0.333
Abreast, $\psi =60^{\circ }$	0.212	0.365	0.229	0.438
Abreast, $\psi =90^{\circ }$	0.235	0.430	0.229	0.421

**Table 7:** Sensitivity to the sea clutter parameters. Group certified denotes the number of the two tested abreast geometries at $\psi =60^{\circ }$ and $90^{\circ }$ satisfying the complete Stage 1 certification criterion. The $P_{\mathrm{exact}}$ range is taken over the four representative geometries, and the maximum LCB is the largest ${\mathrm{LCB}}_{95}\left[P_{N}\right]$ over all tested cardinalities and geometries.

Clutter Condition	Group Certified	$P_{\mathrm{exact}}$ Range	Max. LCB
$\nu =1.18,\;\tau_{c}=13$ ms	2/2	0.138–0.257	0.487
$\nu =0.70,\;\tau_{c}=13$ ms	2/2	0.127–0.233	0.446
$\nu =2.00,\;\tau_{c}=13$ ms	2/2	0.147–0.247	0.504
$\nu =1.18,\;\tau_{c}=10$ ms	2/2	0.152–0.253	0.513
$\nu =1.18,\;\tau_{c}=20$ ms	0/2	0.145–0.215	0.438

**Table 8:** Sensitivity to the PCA dimensionality and covariance shrinkage in Stage 2. Ranges denote the minimum and maximum values across the four representative geometries. PCA variance is averaged over the cross-validation folds.

Parameter	Setting	$P_{\mathrm{exact}}$	Max. LCB	Retained Variance (RA/RD)
PCA components	16	0.153–0.202	0.504	0.624–0.970/0.692–0.988
	32	0.158–0.227	0.463	0.787–0.981/0.771–0.992
	64	0.156–0.252	0.496	0.913–0.992/0.859–0.996
$\lambda_{\mathrm{sh}}$	0.1	0.159–0.253	0.454	–
	0.2	0.157–0.255	0.471	–
	0.4	0.158–0.227	0.463	–
	0.6	0.158–0.230	0.513	–
	0.8	0.156–0.215	0.513	–

## Data Availability

The data presented in this study are available upon request from the corresponding author.

## Abbreviations

The following abbreviations are used in this manuscript:

AESA	Active electronically scanned array
AIC	Akaike information criterion
CFAR	Constant false alarm rate
CI	Confidence interval
CNR	Clutter-to-noise ratio
CPI	Coherent processing interval
CSIR	Council for Scientific and Industrial Research
LCB	Lower confidence bound
LDA	Linear discriminant analysis
MDL	Minimum description length
MFR	Multifunction radar
OS-CFAR	Ordered-statistic constant false alarm rate
PCA	Principal component analysis
PFA	Probability of false alarm
PRF	Pulse repetition frequency
RA	Range–angle
RCS	Radar cross-section
RD	Range–Doppler
RLOS	Radar line of sight
SBL	Sparse Bayesian learning
SNR	Signal-to-noise ratio
UCB	Upper confidence bound
USV	Unmanned surface vehicle
